# Surgical Technique for Imageless Robotic-Assisted Revision Total Knee Arthroplasty

**DOI:** 10.1016/j.artd.2025.101837

**Published:** 2025-09-20

**Authors:** Sebastian Braun, Kristen I. Barton, Brent A. Lanting, James L. Howard

**Affiliations:** a Clinical Fellow in Hip and Knee Adult Reconstruction, Division of Orthopaedic Surgery, Department of Surgery, Schulich School of Medicine and Dentistry, Western University and London Health Sciences Centre, London, Ontario, Canada; b Center for Musculoskeletal Surgery, Charité - Universitätsmedizin Berlin, corporate member of Freie Universität Berlin and Humboldt-Universität Zu Berlin, Berlin, Germany; c Adjunct Research Professor, Faculty of Health Sciences, Division of Orthopaedic Surgery, Department of Surgery, Western University and London Health Sciences Centre, London, Ontario, Canada; d Division of Orthopaedic Surgery, Department of Surgery, Schulich School of Medicine and Dentistry, Western University and London Health Sciences Centre, London, Ontario, Canada; e Division of Orthopaedic Surgery, Department of Surgery, Schulich School of Medicine and Dentistry, Western University and London Health Sciences Centre, London, Ontario, Canada

**Keywords:** Revision total knee arthroplasty, Robotic-assisted surgery, Imageless robotic navigation

## Abstract

Success in total knee arthroplasty (TKA) depends on restoring proper joint alignment and implant positioning. While robotic-assisted systems enhance precision in primary TKA, their use in revision TKA is limited due to challenges like bone loss, soft tissue contractures, and metal artifacts. This manuscript presents an imageless robotic navigation technique for revision TKA, eliminating the need for preoperative imaging and allowing intraoperative flexibility. After registering anatomical landmarks and implant removal, the system reassesses anatomy for iterative adjustments based on bone and soft tissue conditions. Unlike traditional canal-referenced methods, this approach aligns components relative to the joint line, enabling individualized positioning. Real-time feedback guides accurate bone cuts and soft tissue balancing. A case example illustrates the procedure. Further studies are needed to confirm long-term clinical benefits.

## Introduction

Long-term clinical outcomes and implant survivorship in total knee arthroplasty (TKA) heavily depend on the accurate restoration of knee joint alignment, balancing, and the optimal positioning of implant components [1]. Robotic-assisted total joint arthroplasty (TJA) is increasingly being utilized, expecting that such technology will significantly improve the precision of bone cuts and implant positioning, with the goal of ultimately enhancing clinical outcomes and long-term implant survivorship [2,3]. Recent studies have demonstrated satisfying radiological and clinical outcomes with robotic-assisted TJA approaches; however, long-term outcomes remain limited [4,5].

Despite the advantages associated with robotic systems in primary TKA, their use in revision TKA (rTKA) has not been as widespread. This reluctance is largely due to the inherent complexities of revision surgery, which often involves massive bone loss, compromised anatomical structures, poor bone quality, and/or soft tissue contractures—all of which contribute to a heightened risk of complications [[6], [7], [8]]. These factors complicate both preoperative planning and intraoperative execution. There has been a significant rise in the number of TKA procedures performed annually [9] and the reasons for revision surgery include septic and aseptic loosening, instability, polyethylene wear, and osteolysis [10]. Consequently, the burden of rTKA surgery is anticipated to grow alongside the rising numbers of primary TKAs being performed globally.

Given that revision procedures demand accurate preoperative planning and precise intraoperative identification of anatomical landmarks, the advantage of robotic-assisted technology to yield better implant positioning, improved functional scores, and improved clinical outcomes is attractive [2,11,12]. Unfortunately, while the technology has received Food and Drug Administration approval primarily for use in primary TJA procedures, including unicompartmental knee arthroplasty, total hip arthroplasty, and TKA, its application in revision surgery is still considered off-label.

As robotic technology continues to grow in the field of orthopaedic surgery, early reports and case studies have begun to highlight the successful application of robotic systems in rTKA [[11], [12], [13]]. This initial insight has provided a foundation for further exploration of the utility of robotic-assisted technology in enhancing surgical techniques and outcomes. Given the promising initial findings, the objective of this article was to provide a detailed overview of robotic-assisted rTKA, focusing on surgical techniques, challenges, and the current state of literature supporting this innovative approach.

The current robotic systems available (eg, Mako SmartRobotics: Stryker Orthopaedics, Kalamazoo, Michigan, USA; ROSA Knee System: Zimmer Biomet, Warsaw, Illinois, USA; VELYS: DePuy Synthes, Warsaw, Illinois, USA; CORI, Smith & Nephew, Memphis, Tennessee, USA) were designed with primary TKA in mind, which may limit their effectiveness in addressing the specific challenges posed by revision procedures. For instance, pin placement for tracking can be hindered by existing hardware, obstructing optimal positioning. Moreover, achieving adequate exposure during revision surgery can be significantly more challenging. Scar tissue and soft tissue contractures can often restrict access to the surgical site, complicating the ability of the surgeon to accurately digitize the anatomy for robotic navigation. Specifically, metal artifacts from these implants can degrade imaging quality, complicating the precise identification of bony surfaces for image-based robotic navigation systems like computed tomography (CT)-dependent robotic systems.

An essential aspect of robotic-assisted rTKA is the iterative adjustments that may need to be made during surgery. Unlike primary TKA procedures, which often allow for more straightforward preoperative planning and intraoperative flow, revision surgery frequently necessitates real-time modifications based on the condition of the bone (loss) and surrounding tissues encountered during the operation. Furthermore, the implementation of robotic-assisted rTKA can be challenging due to the absence of a dedicated revision program or platform within the respective computer systems, making it difficult to fully leverage the benefits of robotic technology in these complex procedures.

Several technical challenges remain that need to be addressed before robotic-assisted rTKA can become a standard practice. These challenges include optimizing strategies for managing preoperative metal artifacts, refining registration techniques, and increasing the flexibility of the software platforms utilized in these robotic systems. Metal artifacts from previous implants can distort imaging, affecting the accuracy of registration and implant positioning. To mitigate this, advanced image processing techniques, such as metal artifact reduction software, can be used to enhance image clarity. Additionally, intraoperative registration based on direct bone contact, rather than relying solely on preoperative CT scans, could help bypass the effects of metal interference and improve accuracy. Another challenge is the need for flexible registration techniques, particularly when faced with altered anatomy due to prior surgeries. Using additional registration points on the implant or surrounding bone, for example, as seen in the imageless robotic-navigation system this technical note refers to, can help overcome these limitations. Real-time adaptive registration could further improve precision by continuously updating the system as the surgeon removes implants and resects bone, ensuring the plan remains accurate throughout the procedure. The software platforms driving robotic-assisted rTKA surgery also require ongoing optimization to support rTKA applications as current systems are designed for primary TKA and lack specific features for revision surgeries. Developing revision-specific software with customized templating, real-time adaptive algorithms, and enhanced bone-cut planning could better address the complexities of rTKA. Open-source platforms that allow for more flexibility in implant selection and better data integration for visualization would also improve outcomes. Additionally, incorporating artificial intelligence into the software could further enhance preoperative planning and real-time decision-making. Finally, specialized training for surgeons is essential to effectively use these advanced systems and handle issues like registration errors and metal artifacts. Simulation-based training could help bridge the learning curve, enabling surgeons to adapt more quickly to these complex systems. By addressing these challenges through technological improvements and training, robotic-assisted rTKA has the potential to significantly enhance surgical precision and outcomes, offering a more tailored and effective approach to rTKA.

Despite the challenges associated with robotic-assisted rTKA, we present a technique utilizing imageless navigation systems, which facilitate revision surgeries effectively. These systems enhance intraoperative surgical workflow by allowing for real-time adjustments without the need for preoperative imaging, which may be compromised by metal artifacts. Imageless registration eliminates the reliance on traditional imaging techniques like CT scans, instead using real-time joint surface data and kinetic movement information, such as the alignment of the hip and ankle. This approach enables real-time assessment of the ligamentous integrity and balancing issues that may have been part of the indication for the surgery. This also not only reduces the need for preoperative imaging resources but also minimizes patient radiation exposure compared to traditional image-guided robotic-assisted systems. As a result, these advancements offer a promising pathway to improving surgical workflow, enhancing the application of robotic technology, and potentially improving outcomes in rTKA, providing surgeons with the necessary tools to navigate the complexities of revision procedures.

The technique has been implemented in clinical practice since June 2023, with approximately 40 revision cases performed to date. This experience has contributed to the ongoing refinement and optimization of implant positioning, alignment strategies, and intraoperative balancing protocols in the rTKA setting.

**Figure 1:**
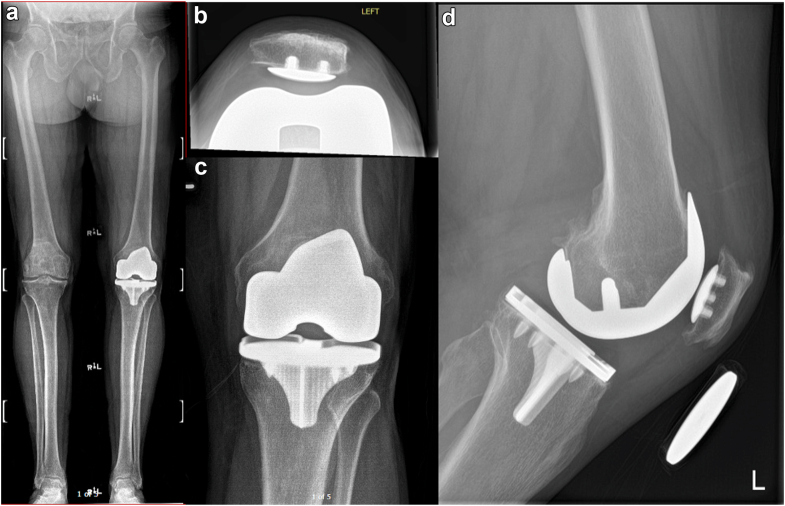
Preoperative radiographs of the left knee prior to rTKA. (a) Long leg standing anteroposterior (AP). (b) Axial patellar view. (c) Knee AP view. (d) Lateral view.

To exemplify the application of the described surgical technique, we present the case of a 62-year-old male with a history of a painful and stiffness following cementless cruciate retaining TKA performed in 2022 at an outside institution, see Figure 1. Written informed consent was obtained from the patient for inclusion of this case in the present report. The patient had persistent pain since the index procedure, with a severely limited range of motion (ROM) (10° to 60°), and substantial functional impairment. After comprehensive clinical and laboratory evaluation ruled out periprosthetic joint infection, the patient underwent rTKA in 10/2024 utilizing the imageless robotic-assisted technique.

Intraoperatively, the procedure included extensive synovectomy, prepatellar bursectomy, implant removal, and reconstruction with a cemented posterior-stabilized femoral component, a tibial cone and cemented tibial revision baseplate, and cemented short stems.

The patient progressed well postoperatively. At 6 weeks, the ROM had already improved to 0° to 100°, and he reported significantly reduced pain. Intraoperative biopsies and cultures were taken demonstrating no signs of low-grade periprosthetic joint infection. By the 3- and 6-month follow-up appointments, the patient achieved further functional gains with improved quadriceps strength and ongoing pain relief.

## Surgical technique

This described concept of imageless robotic-assisted rTKA fundamentally differs from conventional surgical techniques. In traditional approaches, surgeons typically begin the reconstruction of the joint line, only after the removal of the implant. This often involves establishing solid fixation in the tibial diaphysis through long cemented or press-fit cementless stems or in the metaphysis using cones or sleeves, followed by reconstruction of the epiphysis and an estimation of the joint line. Conventional rTKA, relying on intramedullary instrumentation, canal referencing instruments, and canal referencing cutting guides, focuses on fixation based on the medullary canal and subsequent construction to achieve joint line and knee balance surgical goals. In contrast, imageless robotic-assisted rTKA, allows the surgical team to start balancing the rTKA from the defined joint line based on existing implants. The surgeon then adjusts implant position virtually and then optimizes this virtual construct after implant removal in consideration of bone loss. Therefore, the approach starts with consideration of the articulation and extends toward the metaphysis and diaphysis, rather than the reverse.

**Figure 2:**
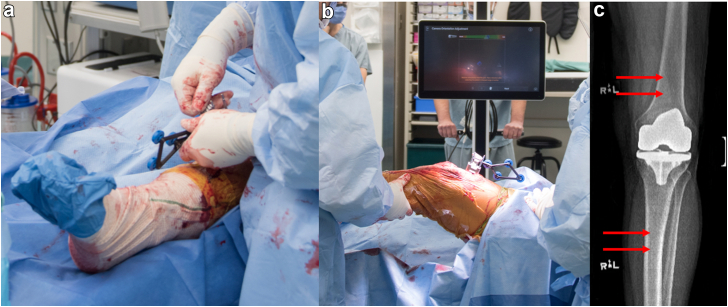
(a) Intraoperative photograph demonstrating the positioning of the tibial array and pins during imageless robotic-assisted rTKA. (b) Intraoperative view showing the placement of the femoral array and the navigation system interface verifying real-time capture and visibility of both arrays. (c) Preoperative radiograph of a left TKA before the rTKA surgery. The pin incisions, marked with red arrows, indicate the pin locations used to guide accurate alignment and component placement during the procedure.

The procedure begins with exposure through a standard medial parapatellar approach, ensuring adequate soft tissue releases to enhance visualization of the surgical field. Careful placement of the femoral and tibial array pins is then performed, allowing for sufficient bone length to accommodate stems while avoiding interference with the removal of previous implants, which is essential for accurate digitization and maintaining the integrity of the navigation system (Fig. 2). For this tibia, this may require separate small incisions distally.

It may be necessary to place femoral pins in the medial condyle if bone conditions permit (Fig. 2B). The tibial array typically requires fixation across the canal, which may require more distal placement to facilitate tibia cone preparation, ensuring that the resection and implant placement can be performed accurately (Fig. 2A). Notably, vigilance in ensuring the tracker arrays are appropriately tight is essential as the increased length of surgery and implant removal may cause loosening of the arrays attachment to the pins and eliminating their utility. In cases without previous stem or metaphyseal fixation methods or rods, the pins can be placed intraincisional, and they can be removed after achieving final tissue balancing and acquiring landmarks after last trial implants, if they do not involve longer-stemmed implants, just prior to the implantation of the final components.

**Figure 3:**
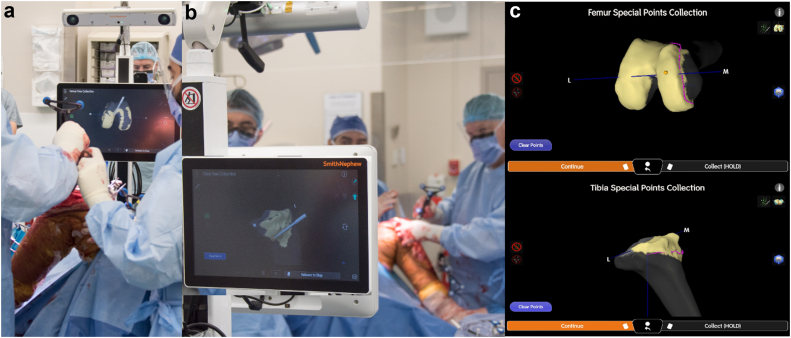
(a) Intraoperative photograph showing the mapping of key anatomical landmarks on the femur using the imageless robotic system during rTKA. (b) Intraoperative photograph demonstrating the tibial mapping process, with the navigation probe identifying specific reference points on the tibial plateau. (c) Screen capture from the navigation system illustrating the completed femoral and tibial point collections. The top of the figure depicts the femoral special points collection, where key anatomical points on the femur are mapped for accurate digitization. The bottom of the figure shows the tibial special points collection, where similar anatomical landmarks on the tibia are recorded. These mappings are crucial for guiding precise bone cuts and implant positioning during the surgery.

Following the placement of pins, the robotic system is utilized to digitize the existing implants, whether they are failed primary TKA components or spacers used during treatment of infection and surrounding bone structures, making necessary adjustments for any anatomical deviations encountered due to prior surgeries. Similarly to the workflow as primary TKA, the system captures key landmarks to ensure precise alignment, including the malleoli, center of the knee, center of the femur, and hip center. Additionally, baseline ROM and knee balance are recorded, and the femoral and tibial surfaces are mapped to guide accurate bone cuts and implant placement, as depicted in Figure 3. This process ensures that the robotic system can adapt to the unique anatomy of each patient, enhancing surgical precision and optimizing outcomes.

**Figure 4:**
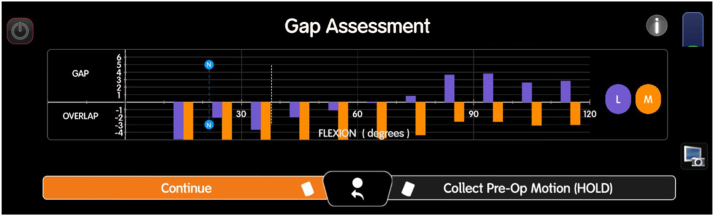
Illustration of the stressed gaps for flexion and extension during robotic-assisted rTKA. The “GAP” represents a loose gap, while “OVERLAP” indicates areas where there is compression. The purple or “L” denotes the lateral side, while the orange or “M” indicates the medial side. The system simulates the behavior of the knee in both flexion and extension to assess soft tissue tension and joint alignment, providing critical data for optimizing implant positioning and balancing the flexion and extension gaps.

After the mapping is completed, the next step in the robotic-assisted rTKA workflow involves using the pre-existing anatomy and ligament tension to guide the flexion and extension gap balancing process. The system first assesses the stressed gaps with the implants in place, simulating how the knee behaves during flexion and extension, see Figure 4.

**Figure 5:**
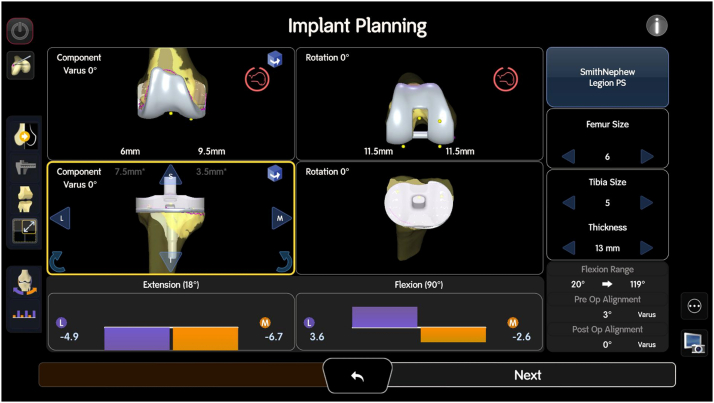
Implant planning interface during robotic-assisted rTKA. The bottom section of the screen displays the stressed ligament tension, with gaps in extension (left) and flexion (right). The purple color represents the lateral compartment, and the orange represents the medial compartment. Numbers below zero indicate a tight gap or overlap, while positive numbers above zero indicate a loose compartment. These data provide critical insight into the existing soft tissue tension and joint alignment.

This provides valuable data on the existing soft tissue tension and the alignment of the joint. The next phase is the poststress starting position, where the robotic system identifies the optimal starting point based on the current joint status, including any deformities or malalignment, see Figure 5.

**Figure 6:**
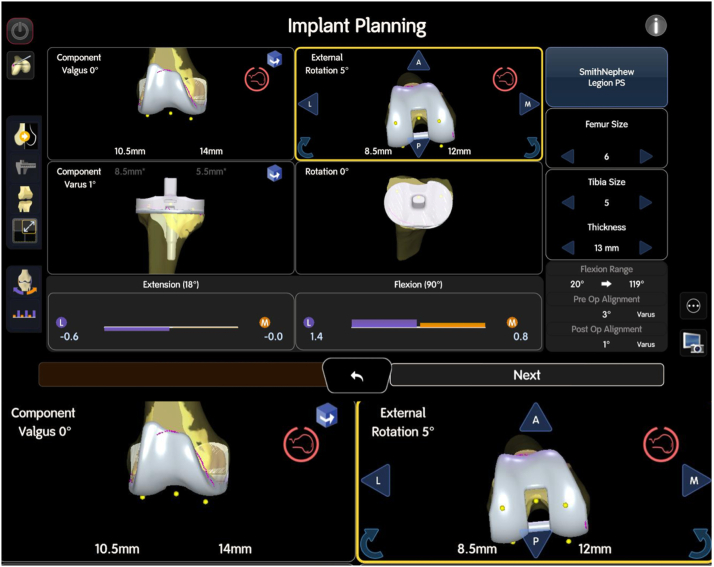
Illustration of balanced flexion and extension gaps in robotic-assisted rTKA. The bottom of the image shows an enlarged view of the femoral cuts, including medial and lateral distal, as well as posterior adjustments, which are crucial for ensuring proper alignment and achieving the desired joint parameters. The surgeon balances the medial and lateral gaps to ensure correct soft tissue tension, while also determining the appropriate bone cuts for the tibial component. This comprehensive planning process establishes the “Home Base” for the final virtual implant positioning, optimizing both alignment and balance before removal of the implant components in place and before the implantation of the final components.

This is followed by the poststressed plan, which defines the final virtual implant positioning and ensures adequate joint parameters are achieved. During this phase, parameters such as the postoperative leg axis and implant sizes are established, along with precise step cuts for the femur, including adjustments for anterior or posterior offset. The system also balances the medial and lateral flexion and extension gaps, ensuring appropriate soft tissue tension. For the tibia, the system evaluates the necessary bone cuts to optimize the placement of the tibial component, see Figure 6. This comprehensive process ensures that both alignment and balance are restored, setting a solid foundation for the implantation of the final components.

**Figure 7:**
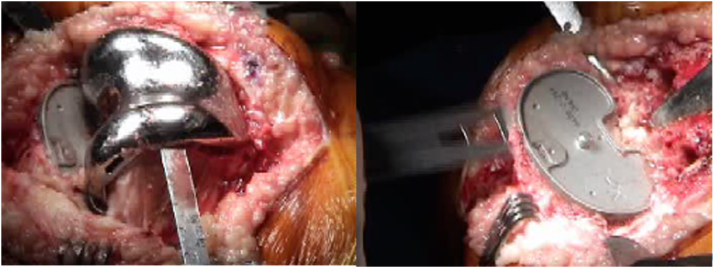
Use of surgical instruments during implant removal in rTKA. On the left, a (flexible) osteotome is being used to carefully detach the previous femoral component from the bone at the cement-component interface. On the right, an oscillating saw is used to remove the tibial component, ensuring precise cuts and minimal bone loss for optimal revision implant placement.

After the femoral and tibial mapping and implant positioning after gap balancing procedure is complete, the next step is the removal of the previous TKA components or articulating spacer. This is done using specialized tools, including an implant removal saw and both flexible and rigid osteotomes, see Figure 7. The implant removal saw is typically used to make precise cuts around the components, while flexible and rigid osteotomes are employed to carefully detach the implants from the surrounding bone. This process is crucial to minimizing bone loss and preserving as much healthy bone as possible for optimal revision implant placement.

Once the old implants are removed, the robotic system can reassess the “new” anatomy, allowing for redigitization or real-time adjustments to ensure accurate knee joint alignment. Notably, each system has different mechanisms to account for the altered anatomy. The CORI system allows additional digitization to account for the bone loss and subsequent adjustment of implant position and optimization of balancing. The bone cuts can then be made in response to the new intraoperative plan. Both the ROSA and VELYS systems do not allow for additional digitization. For these systems, the bone cut plans must be real-time adjusted, with the iterative adjustment of distal femoral and proximal tibial bone cuts and subsequent posterior femoral cut compensation.

**Figure 8:**
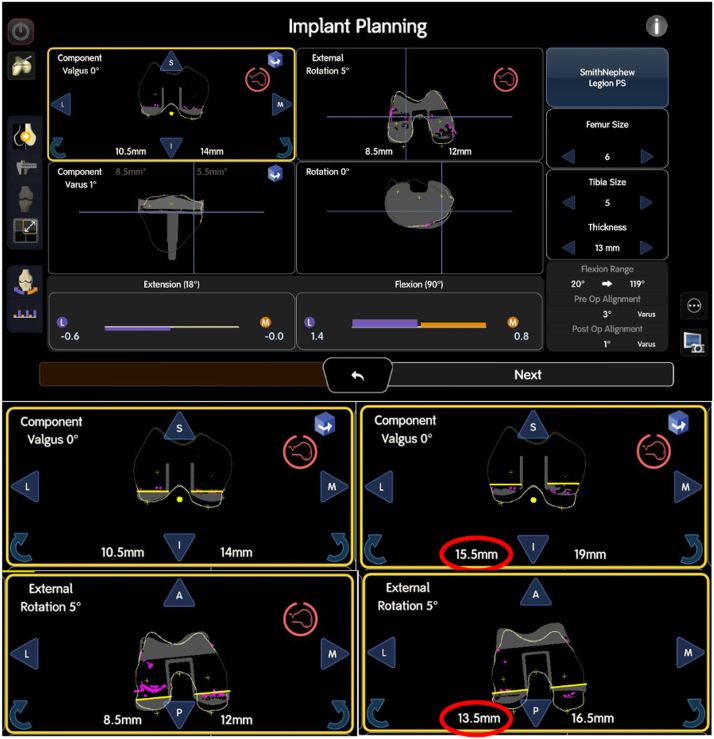
Mapping process after implant removal. On the top row, the small pink dots represent the acquired bone landmark points. In the second row, the distal medial and lateral cuts are illustrated by the yellow lines. The left image demonstrates that the pink dots align with the distal medial cut (14 mm), but not with the distal lateral cut (10.5 mm), indicating bone loss in the lateral distal condyle. The right image shows the adjustment with a +5 mm augment (red circle), bringing the distal lateral cut to 15.5 mm. The bottom row depicts the posterior medial and lateral cuts. The left image shows that the posterior medial cut aligns with the bone landmarks at 12 mm, while the posterior lateral cut displays bone loss, which can be addressed by adding a +5 mm augment, increasing the lateral posterior cut from 8.5 mm to 13.5 mm. These adjustments help compensate for bone loss, ensuring the femoral and tibial components are properly aligned and balanced for optimal joint stability.

**Figure 9:**
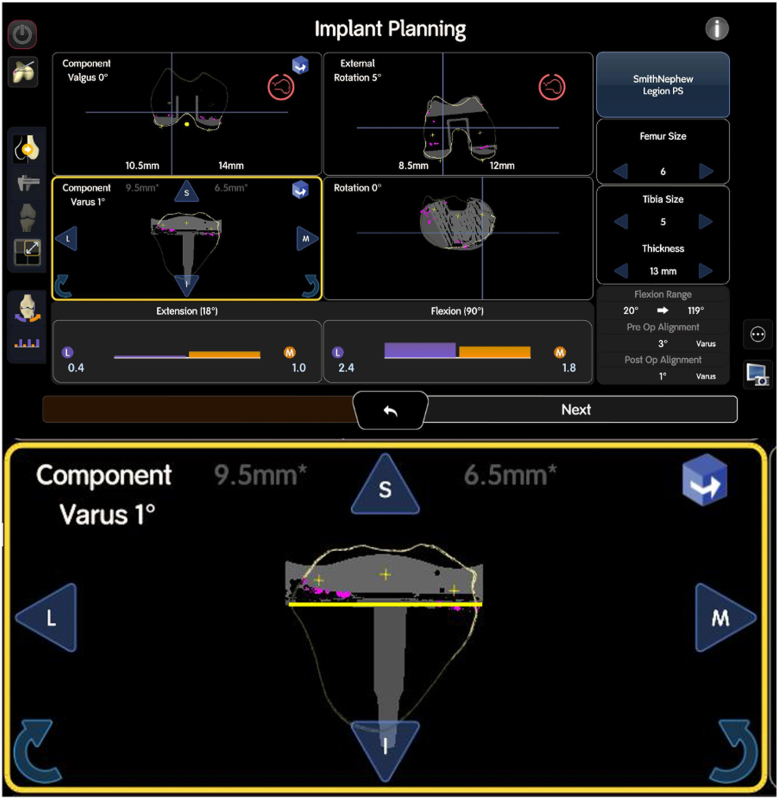
Illustration of the tibial resection process, adjusted inferiorly to align with the lowest special points (in that case medial tibial). The resection (yellow line) catches the most distal landmark/special points (pink dots), ensuring optimal alignment without the need for augments on either side. This step helps in restoring joint stability and optimizing the fit of the implant, with the resection level tailored to match the bone landmarks and achieve proper implant positioning for the revision procedure.

Based on these measurements and the assessment of the remaining bone stock, the surgical plan can be adjusted to include distal medial, lateral, or posterior medial and lateral augments for the femur. The resection level can be lowered in certain increments, depending on the available augment sizes (5 mm in our case example), to compensate for the bone loss and optimize the implant positioning. This same approach applies to the tibia, where adjustments to the resection levels are made to ensure that the implant is placed in the most appropriate position, with additional augments used where necessary, see Figure 8, Figure 9. By revisiting the resection levels and making these adjustments, the surgical team can ensure that both the femoral and tibial components are properly aligned and balanced, accommodating for any deformities and achieving the desired joint stability.

**Figure 10:**
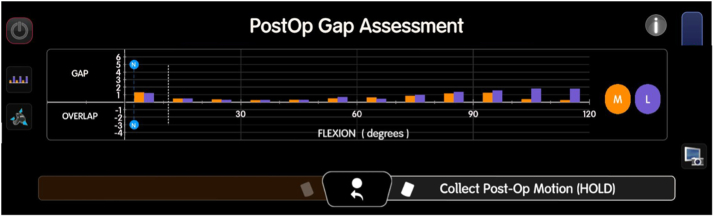
Postoperative stressed gaps with trial implants in place. The gaps are assessed for both flexion and extension, with the lateral compartment represented in purple and the medial compartment in orange. The numbers indicate the gap tightness, with negative values indicating a tight gap and positive values indicating a loose compartment. This stage allows for final adjustments to ligament balancing, ensuring the trial implants provide the desired alignment and stability before final implantation.

Trial components are placed during this process to evaluate ligament balancing and joint stability. This iterative approach is critical for achieving optimal outcomes, as real-time feedback on gaps and ROM is provided by the robotic system, allowing for appropriate soft tissue releases to achieve a well-balanced knee, see Figure 10.

**Figure 11:**
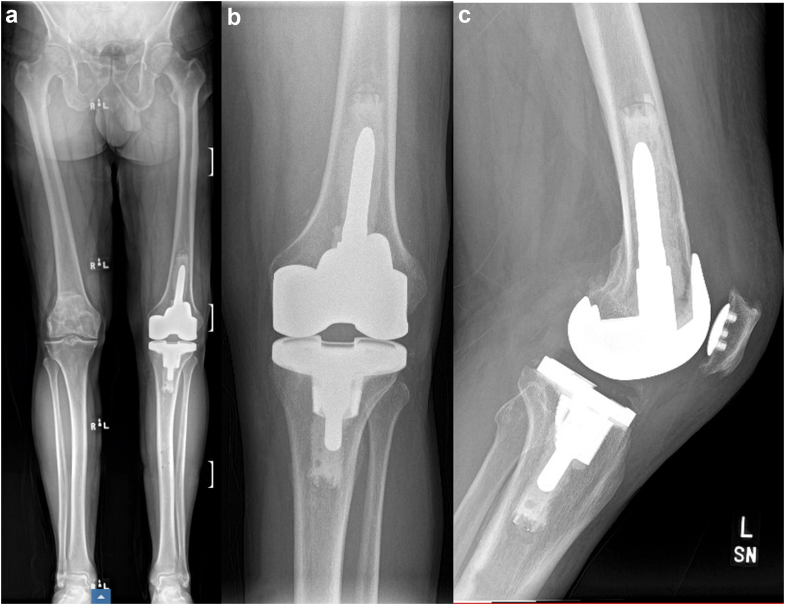
Postoperative radiographs following robotic-assisted rTKA. (a) Full-length standing AP radiograph demonstrating restored mechanical alignment and component positioning. (b) Standard AP view of the knee showing well-aligned femoral and tibial components. (c) Lateral view illustrating proper sagittal positioning and posterior offset of the femoral component. The revision procedure addressed preoperative stiffness, achieving improved joint function and alignment. AP, anteroposterior.

Once the surgeon is satisfied with the trial components and the alignment is determined, the final implantation of the components is carried out, utilizing bone cement or cones and sleeves, if necessary, see Figure 11. Ensuring accurate positioning during this phase is crucial for the long-term success of the revision procedure. The ability of imageless robotic technology to facilitate these processes, without the reliance on potentially compromised imaging from metal artifacts, enhances the feasibility of robotic-assisted revision surgeries. This comprehensive technique highlights the promising role of imageless robotic systems in advancing the effectiveness of rTKA, ultimately hoping to improve patient outcomes.

One of the key advantages of imageless robotic systems is the intraoperative flexibility they offer in defining alignment strategies. In the context of rTKA, where anatomical landmarks are often obscured and soft tissue conditions variable, the ability to individualize implant positioning is particularly valuable. This technique enables the surgeon to select a traditional mechanical alignment target or adopt a more anatomic or personalized alignment strategy, based on the patient’s residual anatomy and intraoperative ligamentous tension.

Importantly, because this approach may facilitate the use of shorter cemented stems rather than relying on long cortical press-fit fixation in the femoral or tibial canals, the surgeon is not constrained by the alignment dictated by diaphyseal reaming. This decoupling from rigid canal referencing allows for greater control over component positioning, enabling alignment decisions to be driven by functional balancing and soft tissue considerations. Real-time data provided by the robotic system support precise adjustments in coronal, sagittal, and rotational planes, allowing the surgeon to balance the knee in a manner tailored to each patient’s needs—particularly valuable in complex revision scenarios.

## Discussion

Incorporating robotic technology into rTKA presents a compelling advantage. The adaptability of robotic systems allows surgeons to adjust their registration strategies dynamically, incorporating additional points on existing implants and surrounding bony surfaces to achieve better knee joint alignment.

Robotic-assisted primary TKA has demonstrated significant improvements in the accuracy and precision of bone cuts and component positioning compared to conventional manual techniques [14,15]. This enhanced precision is especially critical in revision scenarios where the accurate placement of components is essential for successful outcomes (eg, postoperative pain and patient-reported outcome measures or early complications and readmission rates) [16]. Restoration of the joint line to its native level is paramount in both primary and rTKA, yet the lack of clear anatomical landmarks in revision cases often leads to malalignment [17,18]. Traditional surgical techniques that focus on balancing flexion and extension gaps frequently result in an elevated joint line, which can negatively impact functional outcomes [19,20]. The difficulties in achieving a balanced knee after making bone cuts is also a challenge, leading to some advocating for a varus-valgus constrained system for all revision knees.

A previous study by Cochrane et al.'s [16] provides a comprehensive analysis of robotic-assisted rTKA using a second-generation imageless navigation system. This study reviewed 115 patients and focused on short-term outcomes, including readmission rates, complications within 90 days, and patient-reported outcomes. The findings from this cohort demonstrated favorable intraoperative and 90-day postoperative results, including a high percentage of joint line restoration and minimal readmission rates, thus providing compelling evidence that imageless robotic systems are a promising alternative in rTKA [16].

These advancements offered by robotic-assisted techniques may lead to more balanced knees, as these systems provide real-time feedback that enhances the surgeon's ability to achieve optimal knee joint alignment throughout the ROM. These objective data are crucial in ensuring that revisions are conducted with the same level of precision afforded by robotic systems in primary TKA. While initial results of robotic-assisted rTKA are promising, including improved pain scores and the restoration of joint lines, further large-scale studies are necessary to compare long-term outcomes with those of conventional methods. Ongoing developments in robotic technology and the introduction of dedicated software for rTKA will be essential for refining these approaches and validating their efficacy in the complex landscape of knee revision surgery. As the field evolves, the potential for robotic assistance to enhance surgical precision and functional outcomes in rTKA remains a significant area of interest for both surgeons and researchers.

## Summary

Robotic navigation in rTKA represents a growing area of interest, offering the potential for improved precision and outcomes. Despite the challenges inherent in rTKA, the evolving capabilities of robotic systems suggest a promising future for their application in this complex field. Further research is essential to establish standardized protocols and evaluate the long-term benefits of robotic-assisted techniques in the rTKA setting.

## Ethical review statement

Ethics board review was not necessary for this work as it does not constitute a formal study; however, informed consent was obtained from all patients prior to surgery.

## Conflict of interest

K.I. Barton has received royalties from BD Health. B.A. Lanting reports relationships with DePuy, Zimmer Biomet, Stryker, and Smith and Nephew, outside and unrelated to the submitted work. J.L. Howard reports relationships with DePuy, Zimmer Biomet, Intellijoint Surgical, Stryker, A Johnson and Johnson Company, and Smith and Nephew, outside and unrelated to the submitted work; and also is a board member of Hip Society; the other author declares no potential conflicts of interest.

For full disclosure statements refer to https://doi.org/10.1016/j.artd.2025.101837.

## CRediT authorship contribution statement

**Sebastian Braun:** Writing – review & editing, Writing – original draft, Visualization, Validation, Methodology, Investigation, Formal analysis, Conceptualization. **Kristen I. Barton:** Writing – review & editing, Writing – original draft, Visualization, Investigation, Data curation, Conceptualization. **Brent A. Lanting:** Writing – review & editing, Validation, Supervision, Resources, Project administration, Methodology, Investigation, Funding acquisition, Conceptualization. **James L. Howard:** Writing – review & editing, Visualization, Supervision, Resources, Project administration, Investigation, Funding acquisition, Formal analysis, Data curation, Conceptualization.

## Appendix A . Supplementary data

Conflict of Interest Statement for Barton

Conflict of Interest Statement for Braun

Conflict of Interest Statement for Howard

Conflict of Interest Statement for Lanting

## References

[1] Lan R.H., Bell J.W., Samuel L.T., Kamath A.F. (2020). Evolving outcome measures in total knee arthroplasty: trends and utilization rates over the past 15 years. J Arthroplasty.

[2] Wu X.D., Zhou Y., Shao H., Yang D., Guo S.J., Huang W. (2023). Robotic-assisted revision total joint arthroplasty: a state-of-the-art scoping review. EFORT Open Rev.

[3] Deckey D.G., Rosenow C.S., Verhey J.T., Brinkman J.C., Mayfield C.K., Clarke H.D. (2021). Robotic-assisted total knee arthroplasty improves accuracy and precision compared to conventional techniques. Bone Joint J.

[4] Emara A.K., Samuel L.T., Acuña A.J., Kuo A., Khlopas A., Kamath A.F. (2021). Robotic-arm assisted versus manual total hip arthroplasty: systematic review and meta-analysis of radiographic accuracy. Int J Med Robot.

[5] Ng N., Gaston P., Simpson P.M., Macpherson G.J., Patton J.T., Clement N.D. (2021). Robotic arm-assisted versus manual total hip arthroplasty : a systematic review and meta-analysis. Bone Joint J.

[6] Rosso F., Cottino U., Dettoni F., Bruzzone M., Bonasia D.E., Rossi R. (2019). Revision total knee arthroplasty (TKA): mid-term outcomes and bone loss/quality evaluation and treatment. J Orthop Surg Res.

[7] Sculco P.K., Abdel M.P., Hanssen A.D., Lewallen D.G. (2016). The management of bone loss in revision total knee arthroplasty: rebuild, reinforce, and augment. Bone Joint J.

[8] Sculco P.K., Flevas D.A., Jerabek S.A., Jiranek W.A., Bostrom M.P., Haddad F.S. (2024). Management of bone loss in revision total knee arthroplasty: an international consensus symposium. Hss j.

[9] Lewis P.L., Graves S.E., Robertsson O., Sundberg M., Paxton E.W., Prentice H.A. (2020). Increases in the rates of primary and revision knee replacement are reducing: a 15-year registry study across 3 continents. Acta Orthop.

[10] Tarazi J.M., Chen Z., Scuderi G.R., Mont M.A. (2021). The epidemiology of revision total knee arthroplasty. J Knee Surg.

[11] MacAskill M., Blickenstaff B., Caughran A., Bullock M. (2022). Revision total knee arthroplasty using robotic arm technology. Arthroplast Today.

[12] Steelman K., Carlson K., Ketner A. (2021). Utilization of robotic arm assistance for revision of primary total knee arthroplasty: a case report. J Orthop Case Rep.

[13] Ngim H.J., Van Bavel D., De Steiger R., Tang A.W.W. (2023). Robotic-assisted revision total knee arthroplasty: a novel surgical technique. Arthroplasty.

[14] Zhang J., Ng N., Scott C.E.H., Blyth M.J.G., Haddad F.S., Macpherson G.J. (2022). Robotic arm-assisted versus manual unicompartmental knee arthroplasty : a systematic review and meta-analysis of the MAKO robotic system. Bone Joint J.

[15] Zhang J., Ndou W.S., Ng N., Gaston P., Simpson P.M., Macpherson G.J. (2022). Robotic-arm assisted total knee arthroplasty is associated with improved accuracy and patient reported outcomes: a systematic review and meta-analysis. Knee Surg Sports Traumatol Arthrosc.

[16] Cochrane N.H., Kim B.I., Stauffer T.P., Hallows R.K., Urish K.L., Carvajal Alba J.A. (2024). Revision total knee arthroplasty with an imageless, second-generation robotic system. J Arthroplasty.

[17] Laskin R.S. (2002). Joint line position restoration during revision total knee replacement. Clin Orthop Relat Res.

[18] Porteous A.J., Hassaballa M.A., Newman J.H. (2008). Does the joint line matter in revision total knee replacement?. J Bone Joint Surg Br.

[19] Hitt K., Bhowmik-Stoker M., Howard M., Mittal Y., Heekin R.D., Jacofsky D. (2015). Joint line restoration in a contemporary revision knee system. J Knee Surg.

[20] Romero J., Seifert B., Reinhardt O., Ziegler O., Kessler O. (2010). A useful radiologic method for preoperative joint-line determination in revision total knee arthroplasty. Clin Orthop Relat Res.

