# Supplementary material for: Surgical Technique for Imageless Robotic-Assisted Revision Total Knee Arthroplasty
Source: Arthroplast Today. 2025 Sep 20;35:101837. doi: 10.1016/j.artd.2025.101837 (PMC12489787; doi:10.1016/j.artd.2025.101837)
Supplement: Conflict of Interest Statement for Howard [file mmc3.pdf]

INDIVIDUAL CONFLICT OF INTEREST STATEMENT

American Association of Hip and Knee Surgeons

(Adopted from the American Academy of Orthopaedic Surgeons disclosure statement)

The following form **must be filled out completely and submitted by each author (example, 6 authors, 6 forms). All items require a response. If there is no relevant disclosure for a given item, enter "*None.*"**

Precision in Revision Total Knee Arthroplasty: Surgical Techniques in Robotic-Assisted Surgery

Manuscript Title

1. Royalties from a company or supplier (The following conflicts were disclosed)
none

2. Speakers bureau/paid presentations for a company or supplier (The following conflicts were disclosed)
DePuy, A Johnson and Johnson Company, Intellijoint, Smith and Nephew, Stryker

3A. Paid employee for a company or supplier (The following conflicts were disclosed)
none

3B. Paid consultant for a company or supplier (The following conflicts were disclosed)
DePuy, A Johnson and Johnson Company, Intellijoint, Smith and Nephew, Stryker

3C. Unpaid consultants for a company or supplier (The following conflicts were disclosed)
none

4. Stock or stock options in a company or supplier (The following conflicts were disclosed)
none

5. Research support from a company or supplier as a Principal Investigator (The following conflicts were disclosed)
DePuy, A Johnson and Johnson Company

6. Other financial or material support from a company or supplier (The following conflicts were disclosed)
Smith and Nephew, Stryker, Zimmer, DePuy, A Johnson and Johnson Company

7. Royalties, financial or material support from publishers (The following conflicts were disclosed)
none

8. Medical/Orthopaedic publications editorial/governing board (The following conflicts were disclosed)
none

9. Board member/committee appointments for a society (The following conflicts were disclosed)

Hip Society

Each author must sign AND print or type his/her name, date and submit a separate form

In addition, one BLINDED Conflict of Interest form (no author names used) should be submitted per manuscript with all author disclosures.

James Howard	[signature]	January 21, 2025
Author Name (Print or Type)	Author Signature	Date
